# A plasma proteolysis pathway comprising blood coagulation proteases

**DOI:** 10.18632/oncotarget.7261

**Published:** 2016-02-07

**Authors:** Lu Yang, Yun Li, Arup Bhattacharya, Yuesheng Zhang

**Affiliations:** ^1^ Department of Chemoprevention, Roswell Park Cancer Institute, Buffalo, NY, USA; ^2^ Department of Urology, Roswell Park Cancer Institute, Buffalo, NY, USA

**Keywords:** amyloid β1-42, coagulation factor VII, coagulation factor XII, prolidase, proteolysis, Pathology Section

## Abstract

Coagulation factors are essential for hemostasis. Here, we show that these factors also team up to degrade plasma proteins that are unrelated to hemostasis. Prolidase, SRC and amyloid β1-42 (Aβ1-42) are used as probes. Each probe, upon entering the blood circulation, binds and activates factor XII (FXII), triggering the intrinsic and common coagulation cascades, which in turn activate factor VII, a component of the extrinsic coagulation cascade. Activated factor VII (FVIIa) rapidly degrades the circulating probes. Therefore, FXII and FVIIa serve as the sensor/initiator and executioner, respectively, for the proteolysis pathway. Moreover, activation of this pathway by one probe leads to the degradation of all three probes. Significant activation of this pathway follows tissue injury and may also occur in other disorders, e.g., Alzheimer's disease, of which Aβ1-42 is a key driver. However, enoxaparin, a clinically used anticoagulant, inhibits the proteolysis pathway and elevates plasma levels of the probes. Enoxaparin may also mitigate potential impact of activators of the proteolysis pathway on coagulation. Our results suggest that the proteolysis pathway is important for maintaining low levels of various plasma proteins. Our finding that enoxaparin inhibits this pathway provides a means to control it. Inhibition of this pathway may facilitate the development of disease biomarkers and protein therapeutics, e.g., plasma Aβ1-42 as a biomarker of Alzheimer's disease or recombinant human prolidase as an antitumor agent.

## INTRODUCTION

Proteolysis is essential for cells and organisms and serves many purposes. For example, many proteins undergo limited post-translational proteolytic processing in order to become functionally active, while unwanted or abnormal proteins and peptides are promptly degraded to prevent accumulation or aggregation. Two major intracellular proteolysis pathways are well known: proteolysis in lysosome [1], and ubiquitination-dependent or -independent proteolysis in proteasome [2, 3]. Proteolysis also occurs in the plasma, e.g., limited proteolytic processing of blood coagulation zymogens to become functionally active. However, a plasma proteolysis pathway for eliminating unwanted or abnormal proteins has not been previously shown. Various endogenous or exogenous proteins and peptides may enter the blood circulation, some of which may not have any physiological functions in the blood or may even be harmful. The fate of these substances in the plasma is not well understood, although certain plasma proteins may be removed through renal elimination, hepatic elimination or the immune system along with endocytosis and degradation by the intracellular proteolysis pathways [4, 5].

In this paper, we show that blood coagulation factors work in concert to enable proteolysis of non-coagulation factors in the plasma, including prolidase, also known as peptidase D (PEPD), SRC and amyloid β1-42 (Aβ1-42), and discuss clinical implications of this finding. PEPD is mainly an intracellular protein, a dipeptidase involved in collagen metabolism [6]. Interestingly, we recently found that recombinant human PEPD inhibits tumor growth by targeting epidermal growth factor receptor (EGFR), also known as ERBB1, and its family member ERBB2 [7, 8]. SRC is an intracellular non-receptor protein tyrosine kinase [9]. Both PEPD and SRC are present in the plasma at low levels under normal circumstances, as shown in the present study, presumably due to their release from damaged cells and tissues. Aβ1-42 is a peptide generated from proteolytic cleavage of cell-membrane-bound Aβ precursor protein by secretases and is critically involved in the development of Alzheimer's disease (AD) [10]. Aβ1-42 is also present in the plasma at low levels under normal circumstances. PEPD, SRC and Aβ1-42 were evaluated as three probes to show how different substrates may engage the plasma proteolysis pathway and also in view of the potential clinical implications of their degradation in the plasma.

## RESULTS

### PEPD is degraded in the plasma by coagulation proteases but enoxaparin inhibits the degradation

**Figure 1:**
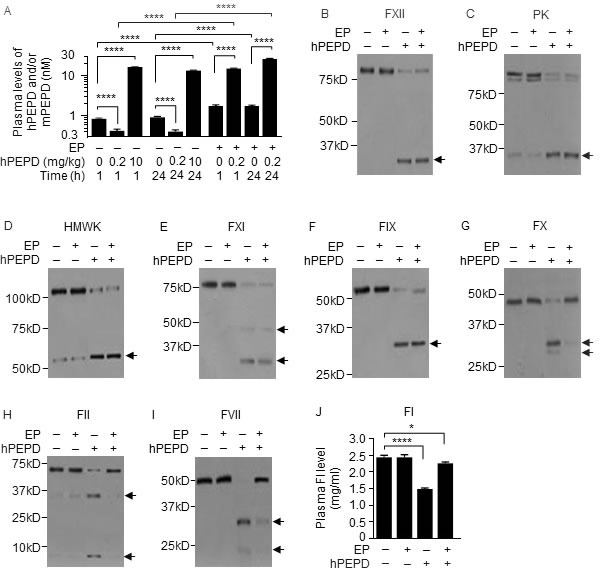
PEPD degradation in the plasma **A.** Plasma PEPD concentrations in WT mice treated with EP and/or hPEPD. EP (2.5 mg/kg) was given to mice i.p. once daily for 5 days. hPEPD (0.2 or 10 mg/kg) or vehicle was given to mice i.p. alone or 1 h after the last EP dose. Blood samples were collected from the mice at 1 or 24 h after hPEPD/vehicle treatment for measurement of plasma PEPD by enzyme-linked immunosorbent assay (ELISA). **B.**-**J.** Changes in plasma coagulation factors in WT mice treated with EP and/or hPEPD. Mice were treated with EP as described in A. hPEPD (0.2 mg/kg) or vehicle was given to mice i.p. alone or 1 h after the last EP dose; blood samples were collected from the mice at 6 h after hPEPD/vehicle treatment; 7.5 μl plasma per sample was analyzed by immunoblotting (IB). Arrows indicate cleaved fragments. Plasma level of FI was measured by ELISA. Error bars in A and J indicate SD (*n* = 3). Data were analyzed by two-way ANOVA in A or one-way ANOVA in J, followed by Tukey multiple comparisons test. Data in A were log transformed before ANOVA. * *p* < 0.05; **** *p* < 0.0001.

Average plasma level of mouse PEPD (mPEPD) was 0.9 nM in control mice, which is very similar to our previously obtained values [11], and average plasma levels of total PEPD (hPEPD plus mPEPD) increased 19.4- and 15.1-fold at 1 and 24 h, respectively, following intraperitoneal injection (i.p.) of recombinant human PEPD (hPEPD) at 10 mg/kg (Figure 1A). Surprisingly, at 1 and 24 h following hPEPD injection at 0.2 mg/kg, average plasma levels of total PEPD were only 51% and 46% of control, respectively (Figure 1A). This suggested that hPEPD might elicit rapid elimination of itself and mPEPD from plasma in mice.

Activators of antithrombin seem to elevate plasma PEPD level in rats [12]. We treated mice with enoxaparin (EP), a clinically used low molecular weight heparin which activates antithrombin III, at 2.5 mg/kg i.p. daily for 5 days and gave hPEPD (0.2 mg/kg) i.p. at 1 h after the last EP dose; average plasma levels of total PEPD were 35.2- and 63.8-fold higher at 1 and 24 h, respectively, following hPEPD treatment than in mice treated with the same dose of hPEPD alone (Figure 1A). EP also increased average plasma level of mPEPD by 2.1-fold (Figure 1A). hPEPD treatment caused cleavage or activation of many coagulation factors in mice, including factor XII (FXII), prekallikrein (PK), high molecular weight kininogen (HMWK), factor XI (FXI), factor IX (FIX), factor X (FX), factor II (FII), factor I (FI) and factor VII (FVII) (Figure 1B–1J). FXII, PK, HMWK, FXI, FIX are components of the intrinsic blood coagulation cascade [13], whereas FVII belongs to the extrinsic blood coagulation cascade, which is typically activated *via* tissue factor (TF) upon blood vessel damage. The two cascades converge on FX, activation of which leads to FII activation, which cleaves FI [14]. EP itself had no effect on these factors and failed to prevent hPEPD from activating FXII, PK, HMWK, FXI and FIX, but prevented hPEPD from causing activation or cleavage of FX, FII, FI and FVII (Figure 1B–1J). EP is known to inhibit FIXa, FXa and FIIa (the active forms of FIX, FX and FII) by activating antithrombin III [15, 16]. Because both FIIa and FXa can activate FVII [17], it suggested that hPEPD activated FVII by activating the intrinsic and common coagulation cascades. Although hPEPD is of human origin and has a His tag, injecting mPEPD without any tag to mice elicited the same response as did hPEPD (Supplementary Figure 1C–1L).

**Figure 2:**
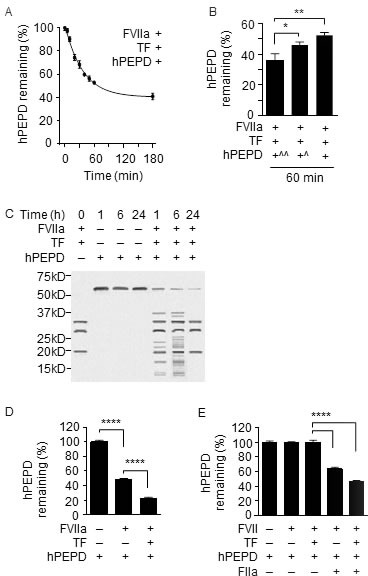
hPEPD degradation by FVIIa **A.** hPEPD (90 nM) was incubated with FVIIa (10 nM) plus TF (10 nM) in CaCl_2_-containing phosphate-buffered saline (PBS) at room temperature (RT), and then measured at different times for remaining hPEPD by enzymatic activity analysis. **B.** hPEPD at 10 nM (+^^), 40 nM (+^) or 90 nM (+) was incubated with FVIIa (10 nM) plus TF (10 nM) in CaCl_2_-containing PBS at RT for 60 min; remaining hPEPD was measured by enzymatic activity analysis. **C.** hPEPD (90 nM) was incubated alone or with FVIIa (10 nM) plus TF (10 nM) in CaCl_2_-containing PBS at RT, and then separated by sodium dodecyl sulfate polyacrylamide gel electrophoresis (SDS-PAGE) and stained by silver. FVIIa and TF were also incubated without hPEPD, as a control. **D.** hPEPD (90 nM) was incubated with FVIIa (10 nM) with or without TF (10 nM) in CaCl_2_-containing PBS at RT, followed by measurement of remaining hPEPD enzymatic activity. **E.** hPEPD (90 nM) was incubated alone, with FVII (10 nM), with FVII (10 nM) plus TF (10 nM), with FVII (10 nM) plus FIIa (100 nM), or with FVII (10 nM) plus TF (10 nM) and FIIa (100 nM) in CaCl_2_-containing PBS at RT for 24 h, followed by measurement of remaining hPEPD by enzymatic analysis. Error bars in A, B, D and E indicate SD (*n* = 3). Data in B, D and E were analyzed by one-way ANOVA, followed by Tukey multiple comparisons test. * *p* < 0.05; ** *p* < 0.01; ****p < 0.0001.

We next evaluated the effects of several human coagulation factors on hPEPD. We focused on FXa, FIIa and FVIIa (active form of FVII), as EP inhibition of PEPD degradation was associated with inhibition of these factors. FXa and FIIa had no effect on hPEPD stability (Supplementary Figure 2A–2D), but FVIIa (10 nM) plus TF (10 nM) caused rapid and extensive hPEPD proteolysis (Figure 2A–2C). For example, only 35.9%, 46.3% and 52.4% of hPEPD remained after it was incubated at 10, 40 and 90 nM with FVIIa and TF for 60 min, respectively (Figure 2B). Notably, given that the normal plasma concentration of FVII is approximately 10 nM [18], the FVIIa concentration used in the above experiment is probably much higher than its physiological concentration, which was intended to facilitate detection of its activity. However, as described later, FVIIa is essential for hPEPD degradation in the plasma. Although TF enhances the proteolytic activity of FVIIa, as expected, it may not necessarily be involved in PEPD degradation by FVIIa *in vivo*, as TF is present in subendothelial tissue, and no blood vessel injury appears to be involved in the activation of FVII and other coagulation proteases in mice injected with hPEPD. While a soluble bioactive form of TF circulates in blood [19], potential involvement of this factor in PEPD degradation by FVIIa remains unknown. Notably, FVIIa in the absence of TF degraded hPEPD (Figure 2D). Moreover, while FVII with or without TF showed no proteolytic activity towards hPEPD, adding FIIa at a physiologically relevant concentration (100 nM) [20] to the mixture caused marked hPEPD degradation (Figure 2E). These results further show that hPEPD activates FVII *via* the intrinsic and common coagulation cascades and also reveal that FVIIa degrades hPEPD. FVIIa is a trypsin-like serine protease, cleaving peptide bonds at the carboxyl side of arginine and lysine [21] and is known to cut FIX and FX. The extensive proteolysis of hPEPD by FVIIa, however, contrasts with its limited proteolysis of FIX (two proteolytic sites) and FX (one proteolytic site) [22, 23]. The proteolytic sites in hPEPD for FVIIa are not yet known.

### hPEPD directly binds and activates FXII

**Figure 3:**
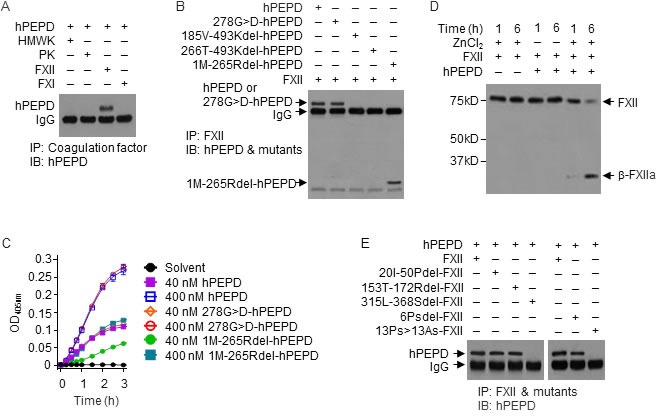
hPEPD binds to PRD in FXII and activates FXII **A.**, **B.** hPEPD or a mutant (40 nM) was incubated with a blood coagulation factor (0.5 μM) in PBS at 37°C for 2 h, followed by immunoprecipitation (IP) and IB. **C.** FXII (0.97 nM) was incubated with hPEPD or a mutant in ZnCl_2_-containing PBS at RT; FXII activation was measured by a chromogenic assay. Error bars indicate SD (*n* = 3). **D.** hPEPD (40 nM) was incubated with FXII (0.5 μM) in PBS (0.1 ml volume) with or without ZnCl_2_ (15 μM) at RT, followed by IB (7.5 μl per sample). **E.** FXII or a mutant (0.5 μM) was incubated with hPEPD (40 nM) in PBS at 37°C for 2 h, followed by IP and IB.

We next evaluated the interaction of hPEPD with several human coagulation factors. FXII interacts with HMWK, PK and FXI to initiate the intrinsic coagulation cascade [24], but hPEPD binds only to FXII (Figure 3A). hPEPD is a homodimeric protein, with each subunit composed of 493 amino acids, containing the N-terminal regulatory domain, a linker and the C-terminal catalytic domain (amino acids #1-174, 175-185 and 186-493, respectively) [25, 26]. We evaluated the interaction of FXII with four hPEPD mutants, including 278G>D-hPEPD, 1M-265Rdel-hPEPD, 185V-493Kdel-hPEPD and 266T-493Kdel-hPEPD (Supplementary Figure 3). The mutants, except 278G>D-hPEPD, cannot form homodimers [7]. Enzymatically inactive 278G>D-hPEPD [27] and 1M-265Rdel-hPEPD were almost indistinguishable from hPEPD in FXII binding, while neither185V-493Kdel-hPEPD nor 266T-493Kdel-hPEPD could bind to FXII (Figure 3B). FXII is a single chain zymogen; proteolytic cleavage at R353-V354 generates FXIIa, with the N-terminal heavy chain and C-terminal catalytic light chain (β-FXIIa) held together by a disulfide bond [28]. hPEPD and 278G>D-hPEPD did not differ in activating FXII, whereas 1M-265Rdel-hPEPD was about 50% active (Figure 3C). These results show: 1) the enzymatic function of hPEPD is not involved in FXII binding and activation; 2) each hPEPD monomer binds to FXII independently; 3) the C-terminal sequence of hPEPD binds to FXII, but additional sequence in hPEPD is involved in full XII activation. Zn^2+^ is involved in FXII activation [29]. FXII activation by hPEPD also required Zn^2+^ (Figure 3D).

We next compared human FXII with its mutants for binding to hPEPD, including 20I-50Pdel-FXII, 153T-172Rdel-FXII, 315L-368Sdel-FXII, 6Psdel-FXII, and 13Ps>13As-FXII (Supplementary Figure 4A–4D). While negatively charged surfaces and other substances activate FXII by binding to sequences within the fibronectin type II domain (FN2D) or fibronectin type I domain (FN1D) in FXII [28], FXII mutants lacking the relevant binding sites (20I-50Pdel-FXII and 153T-172Rdel-FXII) bound to hPEPD as well as did FXII (Figure 3E). However, the FXII mutant lacking the proline-rich domain (PRD; 315L-368Sdel-FXII) could not bind to hPEPD (Figure 3E). Of the 13 prolines in the PRD of FXII (Supplementary Figure 4A), deletion of the first 6 prolines (6Psdel-FXII) only slightly attenuated FXII binding to hPEPD, whereas replacing all 13 prolines with 13 alanines 13Ps>13As-FXII completely abolished its binding to hPEPD (Figure 3E). These results show that hPEPD binds to FXII *via* PRD and that most if not all of the prolines are involved in the binding. This also reveals a physiological function of PRD in FXII.

### FXII initiates PEPD degradation in the plasma

**Figure 4:**
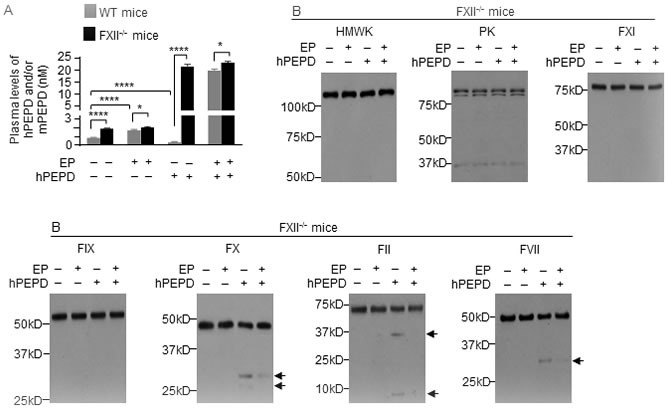
FXII initiates plasma PEPD degradation **A.** Plasma PEPD concentrations in control mice and mice treated with EP, hPEPD, or EP plus hPEPD. EP (2.5 mg/kg) was given to mice i.p. once daily for 5 days; hPEPD (0.2 mg/kg) or vehicle was given to mice i.p. alone or at 1 h after the last EP dose. Blood samples were collected from the mice at 6 h after hPEPD/vehicle treatment for measurement of plasma PEPD level by ELISA. Error bars indicate SD (*n* = 3). Data were log transformed before two-way ANOVA, followed by Tukey multiple comparisons test. *p < 0.05; **** *p* < 0.0001. **B.** Changes in plasma coagulation factors in mice treated as described in A; 7.5 μl plasma per sample was analyzed by IB. Arrows indicate cleaved fragments.

We further studied plasma PEPD degradation using FXII knockout mice (C57BL/6-FXII^−/−^), their WT counterparts, and EP. As expected, FXII was absent in the plasma of FXII^−/−^ mice (Supplementary Figure 5), in which exons 3-8 of the FXII gene is replaced with the neomycin resistance gene [30]. Basal plasma level of mPEPD was 2.2-fold higher in FXII^−/−^ mice than in WT mice (Figure 4A). EP treatment (2.5 mg/kg i.p. daily for 5 days) elevated plasma level of mPEPD by 2.0-fold in WT mice but not in FXII^−/−^ mice (Figure 4A). Without EP pretreatment, at 6 h following hPEPD treatment (0.2 mg/kg i.p.), plasma level of total PEPD decreased 66% in WT mice, as shown before, but increased 12.5-fold in FXII^−/−^ mice (Figure 4A); average plasma level of total PEPD is 53.8-fold higher in FXII^−/−^ mice than in WT mice. With EP pretreatment, at 6 h following hPEPD treatment (0.2 mg/kg i.p.), average plasma level of total PEPD was 19.7 nM in WT mice, which is still 14.6% lower than in FXII^−/−^ mice (Figure 4A), indicating that EP did not fully inhibit FXII-initiated PEPD degradation in WT mice. However, EP did not show a significant effect on plasma PEPD level in FXII^−/−^ mice (Figure 4A). These results show that FXII is essential for degradation of plasma PEPD. In FXII^−/−^ mice, hPEPD treatment caused no activation of HMWK, FXI, PK and FIX, but slight activation of FX, FII and FVII (FI was not measured), which was largely inhibited by EP (Figure 4B). The mechanism of FXII-independent activation of FX, FII and FVII by hPEPD remains unknown, but hPEPD does not directly activate any of these factors (Supplementary Figure 6). Our results do not show that FXII-independent activation of FVII contributes significantly to plasma PEPD degradation. Lack of EP effect on plasma PEPD level in FXII^−/−^ mice, contrary to its ability to markedly elevate plasma PEPD level in WT mice (Figure 1A), also further shows that EP elevates plasma PEPD level in WT mice by inhibiting the FXII-mediated proteolysis pathway, rather than by modulating PEPD exit from plasma, e.g., its sequestration in the matrix or its uptake by cells. Collectively, we conclude that plasma PEPD degradation originates from direct FXII activation by PEPD.

### Plasma PEPD is degraded exclusively by FVIIa

**Figure 5:**
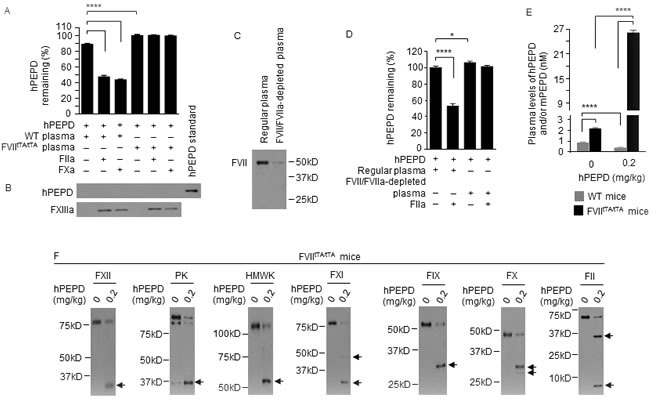
Plasma PEPD is degraded exclusively by FVIIa **A.**, **B.** hPEPD (9.2 pmol) was incubated at RT with plasma (100 μl) with or without FIIa or FXa (10 pmol) for 24 h and after centrifugation to remove the precipitates, remaining PEPD was measured by enzymatic activity analysis. The precipitates were washed by PBS and checked for presence of hPEPD by IB, using FXIIIa and pure hPEPD as a binding control and standard, respectively. **C.** Plasma samples from untreated WT mice were incubated with an antibody that binds to both FVII and FVIIa; the immunocomplexes were pulled down with protein A sepharose. The supernatant fraction along with a regular plasma sample was analyzed for FVII/FVIIa level by IB. FVIIa was undetectable in the samples. **D.** hPEPD (9.2 pmol) was incubated with regular plasma or FVII/FVIIa-depleted plasma (100 μl) in the absence or presence of FIIa (10 pmol) at RT for 24 h; remaining hPEPD was measured by hPEPD enzymatic activity. Notably, plasma samples used in A-D were prepared from blood drawn from mice without an anticoagulant. **E.**, **F.** Plasma PEPD and coagulation factors in control mice and mice at 6 h after hPEPD treatment; PEPD concentration was measured by ELISA, and coagulation factors were analyzed by IB (7.5 μl plasma per sample). Arrows indicate cleaved fragments. Error bars in A, D and E indicate SD (*n* = 3). Data were analyzed by one-way ANOVA in A or two-way ANOVA in D and E, followed by Tukey multiple comparisons test. Data in E were log transformed before ANOVA. * *p* < 0.05; **** *p* < 0.0001.

We next turned to FVII-deficient mice. Replacement of both FVII alleles in mice with a construct containing the tetracycline transactivator (tTA) promoter attached to the FVII cDNA (FVII^tTA/tTA^) results in negligible FVII expression [31]. FVII was undetectable in the plasma of FVII^tTA/tTA^ mice (Supplementary Figure 5). We compared the hPEPD-degrading activities of plasma samples from FVII^tTA/tTA^ mice and WT mice. Notably, blood was drawn from mice without an anticoagulant (EDTA), in order to assess the activities of FVII and other factors, but was immediately centrifuged to remove cells and platelets. Under the same experimental condition, no degradation of hPEPD could be detected after incubation with FVII^tTA/tTA^ plasma, but only 88.8% of hPEPD remained after incubation with WT plasma (Figure 5A), implying the presence of a low level of FVIIa in WT plasma, either preformed or generated during the incubation. Adding human FIIa or FXa to WT plasma markedly enhanced hPEPD degradation, but neither factor was effective in FVII^tTA/tTA^ plasma (Figure 5A). Notably, all incubated solutions were cleared of precipitates (potential fibrin clots) by centrifugation before analysis. The precipitates after wash with PBS were suspended in 2.5% SDS and analyzed by IB for presence of hPEPD, using factor XIIIa (FXIIIa) as the binding control, since FXIIIa is known to bind to fibrin [32]. However, no hPEPD could be detected in any of the samples (Figure 5B), although FXIIIa was also absent in two samples, apparently due to loss of the minute amount of precipitates during PBS wash. Moreover, removal of FVII/FVIIa from WT plasma by immunodepletion abolished the residual hPEPD degradation or FIIa-enabled hPEPD degradation (Figure 5C and 5D). Basal plasma level of mPEPD was 2.5-fold higher in FVII^tTA/tTA^ mice than in WT mice (Figure 5E). At 6 h following hPEPD treatment (0.2 mg/kg i.p.), plasma level of total PEPD decreased 56.3% in WT mice but increased 12.0-fold in FVII^tTA/tTA^ mice, differing by 68.9-fold between the two genotypes (Figure 5E). FVII^tTA/tTA^ mice closely resemble FXII^−/−^ mice with regard to their inability to degrade plasma PEPD, but the coagulation factors that were not activated in hPEPD-treated FXII^−/−^ mice, as shown in Figure 4B, were all activated in hPEPD-treated FVII^tTA/tTA^ mice, excluding FVII (Figure 5F). Collectively, we conclude that plasma PEPD is degraded exclusively by FVIIa and that both FXa and FIIa, formed after FXII activation by PEPD, activate FVII.

### Degradation of plasma SRC by the FXII-FVII proteolysis pathway

**Figure 6:**
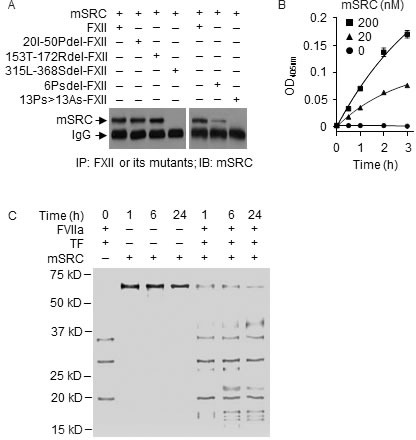
SRC binds to PRD in FXII and activates FXII but is degraded by FVIIa **A.** mSRC (40 nM) was incubated with FXII or a mutant (0.5 μM) in PBS at 37°C for 2 h, followed by IP and IB. **B.** mSRC (0, 20 and 200 nM) was incubated with FXII (0.97 nM) in ZnCl_2_-containing PBS at RT; FXII activation was measured by a chromogenic assay. Each value is mean ± SD (*n* = 3). **C.** mSRC (0.17 μM) was incubated alone or with FVIIa (10 nM) and TF (10 nM) in CaCl_2_-containing PBS at RT for indicated times, separated by SDS-PAGE and stained by silver. FVIIa and TF were incubated without mSRC as a control.

Many proteins bind to proline-rich motifs *via* SH3, WW or EVH1 domains [33–35]. We examined the interaction of SH3-containing mouse SRC (mSRC) with the FXII-FVII pathway. Although mSRC is an intracellular tyrosine kinase, it is present in plasma at a low concentration as shown later, probably due to release from damaged cells. mSRC directly bound and activated FXII (Figure 6A and 6B). Deletion of the PRD in FXII (315L-368Sdel-FXII) completely abolished mSRC binding, but binding was not altered by deletion of the sequences in FN2D or FN1D that binds to negatively charged surfaces and other substances (20I-50Pdel-FXII or 153T-172Rdel-FXII) (Figure 6A). However, mSRC differs from hPEPD in that its binding to FXII was severely reduced after deletion of the first 6 prolines in the PRD of FXII (Figure 6A), whereas such deletion only slightly attenuated hPEPD binding to FXII as described before.

**Figure 7:**
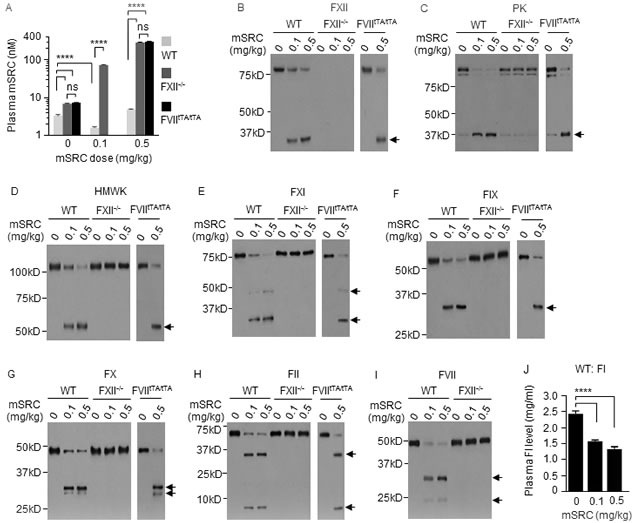
The FXII-FVII proteolysis pathway detects and degrades SRC WT mice, FXII^−/−^ mice and FVII^tTA/tTA^ mice were treated with vehicle or mSRC i.p.; blood samples were collected from the mice at 6 h after treatment. **A.** Plasma concentrations of mSRC, measured by ELISA. **B.**-**J.** Changes in plasma coagulation factors, measured by IB (7.5 μl plasma per sample) or ELISA. Arrows indicate cleaved fragments. Error bars in A and J indicate SD (*n* = 3). Data were analyzed by two-way ANOVA in A or one-way ANOVA in J, followed by Tukey multiple comparisons test. Data in A were log transformed before ANOVA, **** *p* < 0.0001; ns, not significant.

Incubation of mSRC with FVIIa (10 nM) and TF resulted in time-dependent and extensive mSRC fragmentation (Figure 6C); thus, mSRC is also a substrate of FVIIa. The exact proteolytic sites in mSRC for FVIIa are not yet known. Average plasma level of endogenous mSRC was 3.4 nM in WT mice, but was 2.1- and 2.2-fold higher in FXII^−/−^ mice and FVII^tTA/tTA^ mice respectively (Figure 7A). In WT mice, average plasma level of total mSRC was 49% and 147% of control at 6 h post i.p. injection of mSRC at 0.1 or 0.5 mg/kg, respectively (Figure 7A). The effect of the low dose of mSRC on plasma level of total SRC is reminiscent of that seen with low dose of hPEPD as shown in Figure 1A. At 6 h post mSRC injection described above, average plasma level of total mSRC was 45.2-58.2-fold higher in FXII^−/−^ mice and 62.2-fold higher in FVII^tTA/tTA^ mice (treated with mSRC only at 0.5 mg/kg) than in WT mice (Figure 7A). Analysis of plasma samples showed that mSRC injection caused dose-related activation/cleavage of FXII, PK, HMWK, FXI, FIX, FX, FII, FI and FVII in WT mice, no change in any of the factors in FXII^−/−^ mice, and activation of all the factors, excluding FVII, in FVII^tTA/tTA^ mice (Figure 7B–7J). Unlike hPEPD and mPEPD, however, mSRC caused no FXII-independent activation of FX, FII and FVII. We did not measure FI cleavage in FXII^−/−^ mice and FVII^tTA/tTA^ mice. Collectively, our results show that the FXII-FVII pathway also detects and degrades mSRC.

### Degradation of plasma Aβ1-42 by the FXII-FVII proteolysis pathway

**Figure 8:**
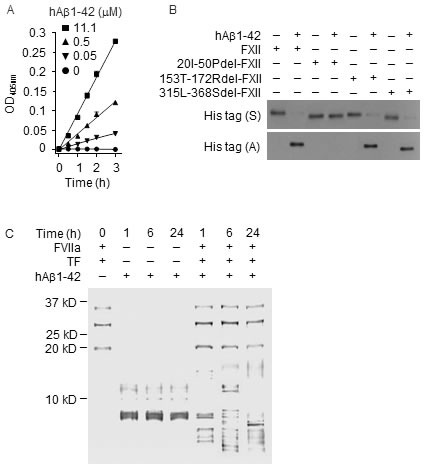
Aβ1-42 binds to FN2D in FXII and activates FXII but is degraded by FVIIa **A.** hAβ1-42 (0, 0.05, 0.5 and 11.1 μM) was incubated with FXII (0.97 nM) in ZnCl_2_-containing PBS at RT; FXII activation was measured by a chromogenic assay. Each value is mean ± SD (*n* = 3). **B.** hAβ1-42 (200 nM) was incubated with FXII or a mutant (20 nM) in PBS at RT; Aβ1-42 aggregated during the incubation. FXII or its mutant remaining in solution (S) or binding to Aβ1-42 aggregates (A), the latter of which was re-dissolved in 2% SDS, were measured by IB. **C.** hAβ1-42 (2.2 μM) was incubated alone, or with FVIIa (10 nM) and TF (10 nM) in CaCl_2_-containing PBS at RT for indicated times; the aggregates were re-dissolved in 2% SDS and mixed with the supernatant fraction, separated by SDS-PAGE and stained by silver. FVIIa and TF were incubated without Aβ1-42, as a control.

human Aβ1-42 (hAβ1-42) also activates human FXII in a dose-dependent manner (Figure 8A), but comparison of hAβ1-42 binding by FXII and its mutants showed that hAβ1-42 binds to FN2D (20I-50P) in FXII (Figure 8B). Incubation of hAβ1-42 with FVIIa (10 nM) and TF resulted in time-dependent and extensive hAβ1-42 fragmentation (Figure 8C). The cleavage pattern indicates that hAβ1-42 is likely cleaved by FVIIa at all three sites where an arginine (residue #5) or a lysine (residues #16, 28) exists. The high molecular weight bands that formed after FVIIa treatment (Figure 8C) may be aggregates of hAβ1-42 or its fragments, as hAβ1-42 is prone to aggregation.

**Figure 9:**
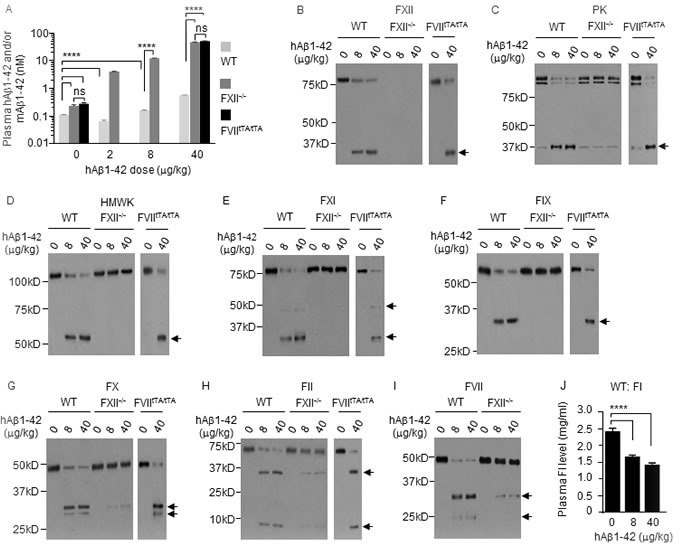
The FXII-FVII proteolysis pathway detects and degrades Aβ1-42 WT mice, FXII^−/−^ mice and FVII^tTA/tTA^ mice were treated with vehicle or hAβ1-42 i.p.; blood samples were collected from the mice at 6 h after treatment. **A.** Plasma concentrations of Aβ1-42, measured by ELISA. **B.**-**J.** Changes in plasma coagulation factors, measured by IB (7.5 μl plasma per sample) or ELISA. Arrows indicate cleaved fragments. Error bars in A and J indicate SD (*n* = 3). Data were analyzed by two-way ANOVA in A or one-way ANOVA in J, followed by Tukey multiple comparisons test. Data in A were log transformed before ANOVA, **** *p* < 0.0001; ns, not significant.

Average plasma level of endogenous mouse Aβ1-42 (mAβ1-42) was 0.1 nM in WT mice, but was 2.2-2.6-fold higher in FXII^−/−^ mice and FVII^tTA/tTA^ mice (Figure 9A). In WT mice, at 6 h after i.p. injection of hAβ1-42 at 2 μg/kg, plasma level of total Aβ1-42 (mAβ1-42 plus hAβ1-42) decreased 39.3%, but it increased 1.5- and 5.1-fold after hAβ1-42 injection at 8 or 40 μg/kg (Figure 9A). The drop in plasma level of total Aβ1-42 in response to the low dose of hAβ1-42 is reminiscent of that seen with low doses of hPEPD or mSRC as described before. Plasma levels of total Aβ1-42 were 62.7-94.1-fold higher in FXII^−/−^ mice and FVII^tTA/tTA^ mice than in WT mice under the same hAβ1-42 treatment, while the difference between FXII^−/−^ mice and FVII^tTA/tTA^ mice was not statistically significant (Figure 9A).

hAβ1-42 caused the activation/cleavage of FXII, PK, HMWK, FXI, FIX, FX, FII, FI and FVII in WT mice, only slight activation of FX, FII and FVII in FXII^−/−^ mice, and activation of all the above factors, excluding FVII, in FVII^tTA/tTA^ mice (Figure 9B–9J). We did not measure FI cleavage in FXII^−/−^ mice and FVII^tTA/tTA^ mice. Our results differ from a previous report, which showed that FXII activation by hAβ1-42 in WT mice leads to activation/cleavage of PK and HMWK but no activation of other coagulation factors [36]. The slight FXII-independent activation of FX, FII and FVII by hAβ1-42 resembles that of hPEPD and mPEPD described before but did not contribute significantly to Aβ1-42 degradation. Although several other proteins were reported to degrade Aβ1-42 elsewhere [37, 38], our results show that Aβ1-42 is degraded exclusively by FVIIa in the plasma *via* the FXII-FVII pathway.

### EP inhibits the degradation of plasma Aβ1-42

**Figure 10:**
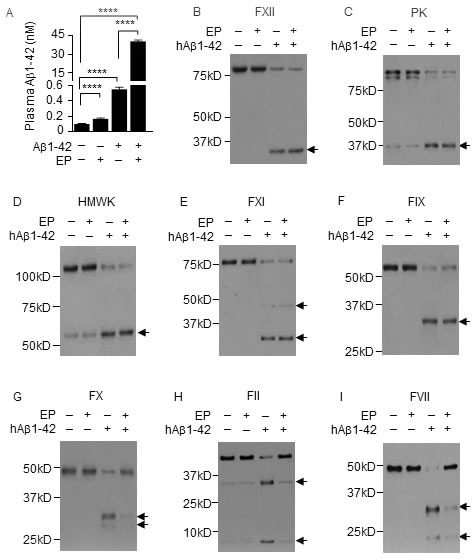
EP inhibits plasma Aβ1-42 degradation and prevents Aβ1-42 from activating FX, FII and FVII **A.** Plasma Aβ1-42 concentrations in control mice and mice treated with EP, hAβ1-42, or EP plus hAβ1-42. EP (2.5 mg/kg) was given to WT mice i.p. once daily for 5 days. hAβ1-42 (40 μg/kg) or vehicle was given to mice i.p. alone or 1 h after the last EP dose. Blood samples were collected from the mice at 6 h after hAβ1-42/vehicle treatment for measurement of plasma Aβ1-42 by ELISA. Error bars indicate SD (*n* = 3). Data were log transformed before one-way ANOVA, followed by Tukey multiple comparisons test. **** *p* < 0.0001. **B.**-**I.** Changes in plasma coagulation factors in control mice and mice treated with EP, hAβ1-42, or EP plus hAβ1-42 as described in A; 7.5 μl plasma per sample was analyzed by IB. Arrows indicate cleaved fragments.

Given that Aβ1-42 is a key driver of AD, we sought to determine whether pharmacologically disrupting the FXII-FVII pathway blocks plasma Aβ1-42 degradation. We treated WT mice with vehicle or EP (2.5 mg/kg i.p.) once daily for 5 days, and 1 h after the last EP/vehicle dose, treated the mice i.p. with hAβ1-42 (40 μg/kg) or vehicle. Blood samples were collected from the mice at 6 h after hAβ1-42/vehicle treatment. Average plasma level of endogenous mAβ1-42 was 0.1 nM in control mice but was 1.7-fold higher in EP-treated mice (Figure 10A). Plasma level of total Aβ1-42 increased 5.5-fold after treatment with hAβ1-42 alone but increased 406.0-fold after treatment with EP plus hAβ1-42 (Figure 10A). Still, EP did not seem to completely block plasma Aβ1-42 degradation, based on the comparison of the above results with that in FXII^−/−^ mice and FVII^tTA/tTA^ mice that received the same hAβ1-42 treatment without EP as described before (Figure 9A).

EP itself had no effect on any of the FXII-FVII pathway factors measured, but greatly inhibited the activation of FX, FII and FVII in hAβ1-42-treated mice (Figure 10B–10I). These results are similar to that shown in EP inhibition of plasma PEPD degradation as described before.

### Activation of the FXII-FVII proteolysis pathway during tissue injury and its protective function

**Figure 11:**
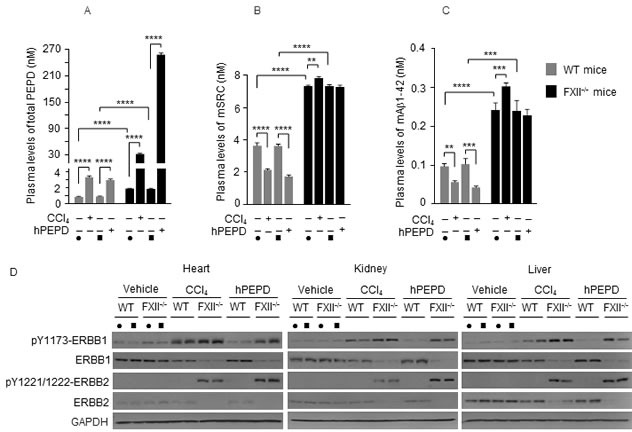
The FXII-FVII proteolysis pathway responds to tissue injury Mice were treated i.p. with vehicle (filled circle: corn oil for CCl_4_; filled square: PBS for hPEPD), CCl_4_ (0.5 g/kg) or hPEPD (4 mg/kg); blood samples and various organs were collected 24 h later. **A.**-**C.** Plasma levels of mPEPD and/or hPEPD, mSRC and mAβ1-42 were measured by ELISA. Error bars indicate SD (*n* = 3); data were log transformed before two-way ANOVA, followed by Tukey multiple comparisons test. ** *p* < 0.01; *** *p* < 0.001; **** *p* < 0.0001. **D.** Tissue levels of ERBB1 and ERBB2 and their phosphorylation status were measured by IB. glyceraldehyde-3-phosphate dehydrogenase (GAPDH) is a loading control. Each lane represents a sample from a different mouse.

Carbon tetrachloride (CCl_4_) causes liver damage and increases serum PEPD level, which presumably is due to PEPD release from the damaged tissues [11, 39]. CCl_4_ treatment (0.5 g/kg) caused significant but similar liver damage between WT mice and FXII^−/−^ mice (Supplementary Figure 7). Plasma level of mPEPD was 2.1-fold higher in FXII^−/−^ mice than in WT mice before CCl_4_ treatment, and at 24 h after CCl_4_ treatment, plasma level of mPEPD increased 3.7-fold in WT mice but increased 16.7-fold in FXII^−/−^ mice (Figure 11A). To corroborate the mPEPD results discussed above, we treated mice with hPEPD at 4 mg/kg. This dose of hPEPD was chosen so that plasma level of total PEPD in hPEPD-treated WT mice is similar to that in CCl_4_-treated WT mice. At 24 h following hPEPD treatment, plasma level of total PEPD increased 3.3-fold in WT mice but increased 134.6-fold in FXII^−/−^ mice (Figure 11A). Thus, whether PEPD is released from damaged tissues or introduced exogenously, it activates the FXII-FVII pathway, which in turn degrades the PEPD. In WT mice treated with CCl_4_ or hPEPD, average plasma levels of mSRC and mAβ1-42 decreased 41.2-56.1% (Figure 11B and 11C). In contrast, in FXII^−/−^ mice, average plasma levels of mSRC and mAβ1-42 increased 6.7% and 24.9%, respectively, after CCl_4_ treatment but remained unchanged after hPEPD treatment (Figure 11B and 11C). The small increase in plasma levels of mSRC and mAβ1-42 in CCl_4_-treated FXII^−/−^ mice may be due to their release from the damaged tissues. Given that these substances are expressed in a variety of organs and tissues, PEPD in particular [40, 41], the above results suggest that injury in various organs and tissues may activate the FXII-FVII pathway *via* release of these and other FXII activators to blood circulation. Furthermore, activation of this pathway by one protein may lead to degradation of multiple plasma proteins that are substrates of FVIIa.

PEPD is a ligand of ERBB1 and ERBB2 which are cell surface receptors. We have recently shown that hPEPD binds to ERBB1 and ERBB2 and causes receptor phosphorylation, followed by profound receptor depletion due to its internalization and degradation, resulting in overall inhibition of receptor activity [7, 8, 11]. We sought to determine whether the FXII-FVII pathway may minimize the inhibitory effects of plasma PEPD (released from damaged tissues or entered exogenously) on the receptors. Compared to FXII^−/−^ mice, WT mice treated with CCl_4_ or hPEPD showed not only lower plasma levels of PEPD (Figure 11A) but also reduced changes in receptor tyrosine phosphorylation (measuring representative phosphorylation sites) and receptor depletion in various tissues (heart, kidney and liver) (Figure 11D). Thus, the FXII-FVII pathway apparently attenuates the inhibitory effect of PEPD on ERBB1 and ERBB2 in normal tissues by degrading plasma PEPD.

## DISCUSSION

**Figure 12:**
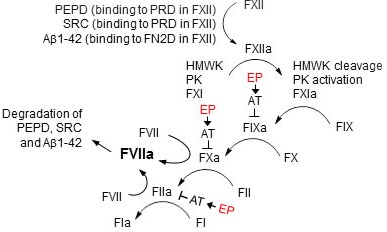
The FXII-FVII proteolysis pathway that detects and degrades PEPD, SRC and Aβ1-42, and its inhibition by EP PEPD, SRC or Aβ1-42 activates FXII by binding to a different domain in FXII. FXII activation leads to activation of FX and FII, which in turn activates FVII, and activated FVII degrades PEPD, SRC and Aβ1-42. EP blocks the degradation of PEPD, SRC and Aβ1-42 in the plasma by binding and activating antithrombin III (AT), which inhibits several coagulation factors in the proteolysis pathway. The “↓” and “T” symbols indicate activation and inhibition, respectively.

The FXII-FVII pathway for detecting and degrading PEPD, SRC and Aβ1-42 as well as its inhibition by EP are summarized in Figure 12. FVIIa degrades PEPD, SRC and Aβ1-42 that are structurally diverse. A previous study also suggests low substrate specificity of FVIIa [21]. PEPD and SRC activate FXII by binding to its PRD, whereas Aβ1-42 activates FXII by binding to its FN2D. Notably, the concentrations of these probes used for FXII activation *in vitro* are based on their concentrations detected in the plasma of FXII^−/−^ mice and FVII^tTA/tTA^ mice following treatment with them; the proteolysis pathway is disrupted in these mice, unlike WT mice in which this pathway was strongly activated under the same treatment conditions, leading to degradation of the probes. Other known activators of FXII interact with FN2D or FN1D in FXII [36, 42, 43]. Thus, multiple domains in FXII mediate its activation. A large number of intracellular proteins bind to proline-rich motifs *via* their SH3, WW or EVH1 domains [33–35]. Our SRC results suggest that other PRD-binding intracellular proteins may also engage the proteolysis pathway when released into blood circulation. On the other hand, it is possible that other mechanisms may also play a role in clearing these and other proteins and peptides from the circulation, such as renal elimination, hepatic elimination, binding to matrix in various tissues as well as cellular uptake and degradation. Notably, there is only slight activation of various factors of the FXII-FVII pathway in un-treated mice (Supplementary Figure 8), suggesting that this pathway is not significantly activated under normal conditions; detection of such activation was somewhat difficult, as there appeared to be a slight activation of the factors following blood draw before the anticoagulant effect of EDTA kicked in.

Our results indicate that the FXII-FVII pathway may contribute to tissue homeostasis by eliminating unwanted or harmful proteins and peptides from plasma, whereas anticoagulants like EP may elevate plasma level of these substances by disrupting the proteolysis pathway. Notably, no physiological function in the blood is known for PEPD, SRC and Aβ1-42, and Aβ1-42 is even harmful, being a key driver of AD. However, our results also show that activation of this pathway leads to significant cleavage of FI, which may increase blood clot risk, although there was no sign of blood clotting in the mice in the present study. Moreover, activation of this pathway may also lead to bradykinin liberation from HMWK and complement activation *via* β-FXIIa and kallikrein, potentially impacting vascular physiology, immune response and inflammation [44–46]. Further study is needed to better understand the protective as well as adverse impact of activation of this pathway.

Our finding that plasma Aβ1-42 is detected and degraded by the FXII-FVII pathway provides insight into AD. Previous studies have shown activation of FXII and certain downstream factors by Aβ1-42 in AD [47, 48], but our results show that such activation leads to FVII activation and degradation of Aβ1-42 by FVIIa. Aβ1-42 and Aβ1-40 are the main components of senile plaque, one of the hallmarks of AD pathology, and are considered key drivers of AD [10, 49]. Like Aβ1-42, Aβ1-40 is generated from proteolytic cleavage of cell-membrane-bound Aβ precursor protein by secretases and is also present in the plasma at low levels. Aβ1-40 may also be degraded by the FXII-FVII pathway, as it differs from Aβ1-42 by only two amino acids, and Aβ1-40 also binds to FXII *via* FN1D [43]. Several other mechanisms of Aβ clearance have been reported, including Aβ internalization by astrocytes [50], Aβ degradation by extracellular or intracellular proteases [38], lowering brain Aβ by peripheral sink such as lipoprotein receptors [51], and apolipoprotein E-mediated Aβ clearance from the brain [52]. However, the fact that total plasma levels of Aβ1-42 are 63-95-fold higher in mice deficient in either FXII or FVII or in EP-pretreated WT mice than in EP-untreated WT mice following hAβ1-42 injection (Figure 9A and 10A) is a clear evidence that the newly discovered plasma proteolysis pathway plays a pivotal role in plasma Aβ1-42 clearance.

Moreover, our finding that EP inhibits plasma Aβ1-42 degradation may provide a novel approach for development of AD biomarkers. Blood-based biomarkers of AD are an unmet medical need. Plasma Aβ level correlates poorly with brain plaque burden and is not currently considered an AD biomarker [53–56]. Our results suggest that temporary inhibition of the FXII-FVII proteolysis pathway, e.g., using EP, may enable detection of high plasma levels of Aβ1-42 and other Aβ forms in AD, and that such an approach may enable development of plasma Aβ as an AD biomarker for disease detection as well as for assessment of disease progression and response to treatment. Interestingly, there is also evidence that EP is a potential therapeutic agent for AD as well. Chronic treatment of AD transgenic mice (APP23 mice or APPswe/PS1dE9 mice) with EP (approximately 2.5 mg/kg, 2-3 times per week) for 3-6 months reduces astrocyte activation and slows disease progression [57, 58]; surprisingly, when EP treatment was started at 5-6 months of age, it reduced brain Aβ accumulation in these mice, although it increased brain Aβ accumulation when EP treatment was started at 10-12 months of age. It remains unclear whether EP blockage of Aβ degradation in the plasma may impact brain Aβ accumulation. Notably, plasma Aβ level was not measured in the studies mentioned above. The therapeutic activity of EP against AD may result in part from its inhibition of the plasma proteolysis pathway, as this pathway may be strongly activated in AD transgenic mice, which may promote blood clotting. Indeed, a recent study showed increased blood clotting activity in the brain of AD transgenic mice [59]. Interestingly, EP was also shown to significantly attenuate Aβ cytotoxicity in cultured neuronal cells.

Finally, the discovery of the FXII-FVII proteolysis pathway may also have important implications for developing certain protein therapeutics. Many therapeutic proteins have short plasma half-life, and current approaches are aimed at slowing their removal *via* biliary, hepatic or renal elimination [4]. Our findings raise the possibility that some of these proteins may engage the FXII-FVII pathway and that inhibiting this pathway may increase their retention in plasma. As a case in point, EP inhibits PEPD degradation *in vivo* and allowed hPEPD dose to be reduced by at least 50 fold without decreasing its plasma concentration required for inhibition of ERBB2-driven tumors in mice [8]. Moreover, combination of EP with hPEPD may also make hPEPD a safer antitumor agent, as EP may minimize the stimulating effect of hPEPD on blood coagulation.

## MATERIALS AND METHODS

### Materials

hPEPD and its mutants (6xHis tagged to the carboxy terminus) were generated, purified and characterized as recently reported [7, 11]. mPEPD was purified from mouse kidney (Supplementary Methods; Supplementary Figure 1A and 1B). hAβ1-42 (A9810) and CCl_4_ were purchased from Sigma-Aldrich. mSRC (50311-M20B)and EP were purchased from Sino Biological and Sanofi-Aventis, respectively. The following human coagulation factors were purchased from Haematologic Technologies: FXII (HCXII-0155), FXI (HCXI-0150), FX (HCX-0050), FXa (HCXA-0060), FVII (HCVII-0030), FVIIa (HCVIIA-0031), TF (RTF-0300), FII (HCP-0010), FIIa (HCT-0020). Human PK (HPK 1302) and human HMWK (HK 1300) were purchased from Enzyme Research Laboratories. The following antibodies were purchased from Santa Cruz Biotechnology: Anti-6XHis tag (sc-803), anti-HMWK (sc-25885), anti-FII (sc-16972), anti-SRC (sc-8056), anti-Aβ1-42 (sc-374527, sc-9129), and a donkey anti-goat IgG-horseradish peroxidase (HRP) (sc-2020). The following antibodies were purchased from Cell Signaling Technology: Anti-ERBB1 (2232), anti-pY1173-ERBB1 (4407), anti-ERBB2 (2165), anti-pY1221/1222-ERBB2 (2243), and anti-SRC (2123). The following antibodies were purchased from GeneTex: Anti-FXII (GTX21008), anti-FXI (GTX79765), anti-FIX (GTX79802), anti-FX (GTX110300), and anti-FVII (GTX79785). Anti-PEPD (ab86507) and anti-GAPDH (MAB374) were purchased from Abcam and Millipore, respectively. A donkey anti-rabbit IgG-HRP (NA934V) and a sheep anti-mouse IgG-HRP (NA931V) were purchased from GE Healthcare.

### Generation of recombinant human FXII and its mutants

Recombinant human FXII and its mutants with 6XHis C-terminal tag (Supplementary Figure 4A and 4B) were generated as described below. The full length human FXII coding sequence without a stop codon was amplified by PCR from normal human liver cDNA using the primers shown in Supplementary Table 1, which contain unique restriction sites. The PCR conditions were as follows: 95°C for 3 min, 29 cycles of 94°C for 30 seconds, 61°C for 30 seconds, and 68°C for 2 min, with a final extension at 68°C for 10 min. Amplified PCR products were digested with applicable restriction enzymes (*Eco*R1 and *Sal*I), followed by ligation into pCMV6-XL5 (Origene), which was pre-digested with the same restriction enzymes. 6XHis C-terminal tag was added to the expression construct by PCR-based site-directed mutagenesis using the primers listed in Supplementary Table 1. The insert was confirmed by DNA sequence analysis. Mutation of proline to alanine as well as specific deletion in the FXII coding sequence, including 20I-50Pdel-FXII, 153T-172Rdel-FXII, 315L-368Sdel-FXII, 6Psdel-FXII, and 13Ps>13As-FXII, were accomplished using QuikChange Lightning Multi Site-Directed Mutagenesis Kit or QuikChange Lightning Site-Directed Mutagenesis Kit (Agilent Technologies), using primers listed in Supplementary Table 1. All the reactions were carried out according to the manufacturer's instructions. All constructs were sequenced to ensure correct changes. These plasmids were used to generate the recombinant proteins in CHO-K1 cells (ATCC). CHO-K1 cells were cultured in F-12K medium (Gibco) supplemented with 10% FBS (Gibco) in a humidified incubator at 37°C with 5% CO_2_. Cells growing in 6-well plates were transfected with the pCMV6-XL5 plasmids expressing FXII or a mutant as described above, using FuGENE HD (Promega), at 1-2 μg of DNA per well for 48 h. Cells were then harvested, washed with PBS and suspended in a lysis buffer (50 mM NaH_2_PO_4_, 300 mM NaCl, 10 mM imidazole, 0.05% Tween 20, with pH adjusted to 8.0 using NaOH) at 0.5 ml per 10^7^ cells. Cell lysis was enhanced by sonication on ice. The lysates were cleared of debris by centrifugation at 10,000 x g for 10 min at 4°C. The 6XHis-tagged FXII and its mutants were purified by Ni-NTA agarose chromatography. The relative molecular size of each protein was checked by IB (Supplementary Figure 4C), and high purity of each protein was confirmed by sodium dodecyl SDS-PAGE and silver staining, using a kit (LC 6070) from Invitrogen (Supplementary Figure 4D). Protein concentrations of all samples were measured by the bicinchoninic acid (BCA) protein assay kit (Pierce).

### Animal studies

All animal studies were performed in accordance with protocols approved by the Institutional Animal Care and Use Committee at Roswell Park Cancer Institute. Male mice at 7-8 weeks of age were used, including wild type (WT) mice (C57BL/6), and mice deficient in FXII (C57BL/6-FXII^−/−^) or FVII (C57BL/6-FVII^tTA/tTA^). WT C57BL/6 mice were purchased from Taconic. C57BL/6-FXII^−/−^ mice and C57BL/6-FVII^tTA/tTA^ mice were bred in our own facility and genotyped as previously described [30, 31]. The breeders were kindly provided by Dr. Francis J. Castellino at University of Notre Dame. All treatments were given by i.p. as follows: A single dose of vehicle, hPEPD, mPEPD, mSRC, hAβ1-42 or CCl_4_; EP once daily for 5 days, followed at 1 h after the last dose of EP with a single dose of vehicle, hPEPD or hAβ1-42. CCl_4_ was dissolved in corn oil, whereas all other substances were dissolved in PBS. The vehicle or the test substance was given to mice in 0.1 ml volume per 20 g body weight. Blood was collected from the mice at specific times by cardiac puncture at the time of sacrifice by carbon dioxide, and heart, liver and kidney were also collected from some of the mice. Blood was collected into K3 EDTA-containing tubes (Multivette 600 from Sarstedt), unless specified otherwise. All blood samples were promptly centrifuged to obtain plasma samples.

### Measurement of plasma levels of PEPD, SRC, Aβ1-42 and FI

Plasma concentrations of hPEPD, mPEPD, mSRC, hAβ1-42, mAβ1-42 and FI (also known as fibrinogen) were determined by ELISA. ELISA for measuring plasma concentrations of hPEPD and mPEPD was described previously [11]. To measure plasma levels of mAβ1-42, total Aβ1-42 (mAβ1-42 plus hAβ1-42) or mSRC, 96-well ELISA plates were coated with an anti-Aβ1-42 mouse monoclonal antibody (sc-374527) or an anti-SRC mouse monoclonal antibody (sc-8056) at 0.25 μg/100 μl/well at 4°C overnight. The plates were washed three times with phosphate buffered saline tween-20 (PBST) and the coated wells were blocked by incubation with 200 μl/well of 1% BSA in PBS for at least 2 h at RT. After another round of wash with PBST, the plates were incubated with appropriately diluted Aβ1-42 standard, SRC standard or plasma samples (100 μl/well) for 2 h at RT. The plates were then washed with PBST and incubated with an anti-Aβ1-42 rabbit polyclonal antibody (sc-9129) or anti-SRC rabbit monoclonal antibody (2123) at 100 μl/well for 2 h at RT. The plates were washed again with PBST, and each well was incubated with a goat anti-rabbit IgG-HRP conjugate (100 μl) for 1 h at RT. After yet another round of wash with PBST, each well was incubated with 100 μl of HRP substrate 3,3′,5,5′-tetramethylbenzedine (Cell Signaling, 7004). Upon adequate color development, 100 μl of stop solution (Cell Signaling, 7002) was added to each well, and absorbance at 450 nm was promptly recorded by a microtiter plate reader. Plasma FI concentration was determined using an assay kit (400-374-130050) from GenWay Biotech, following the manufacturer's instruction.

### IB

Tissue samples were mixed with RIPA buffer (25 mM Tris-HCl, pH 7.6, 150 mM NaCl, 1% Nonidet P-40, 1% sodium deoxycholate, 0.1% SDS), supplemented with 2 mM phenylmethanesulfonyl fluoride, a proteinase inhibitor mix (Roche Applied Science) and phosphatase inhibitor Cocktail 2 (Sigma-Aldrich). Tissue samples were stroked in a Dounce homogenizer, and the homogenates were cleared by centrifugation at 12,000 x g for 15 min at 4°C. Protein concentrations in all samples were measured by the BCA assay kit. Plasma samples were used without further processing (20 μl per sample). Each sample was mixed with 4x loading dye, heated for 5 min at 95°C and then resolved by SDS-PAGE (8-12.5%). Notably, 7.5 μl of original plasma per sample was analyzed. The proteins were transferred to polyvinylidene fluoride membrane, probed with specific antibodies and detected using the ECL Plus Kit (Amersham) or the SuperSignal West Pico Kit (Thermo Scientific).

### Measurement of *in vitro* degradation of hPEPD, mSRC and hAβ1-42

To determine whether a blood coagulation factor degrades hPEPD, hPEPD at 90 nM or lower concentrations (10 or 40 nM) was incubated with solvent, 100 nM FIIa, 100 nM FXa, 10 nM FVIIa with or without 10 nM TF, or 10 nM FVII with or without 10 nM TF and/or 100 nM FIIa in PBS in a total volume of 100 μl containing 5 mM CaCl_2_ for desired times at RT. Notably, TF was solubilized in 10 mM CHAPS, which was diluted by 10-fold in the final assay. To compare the hPEPD-degrading activities of different plasma samples, 9.2 pmol of hPEPD was incubated with 100 μl of plasma from WT mice or FVII^tTA/tTA^ mice at RT, with or without FIIa or FXa (10 pmol), and to maximize detection of any activity, all incubations lasted for 24 h. Notably, blood was drawn into plastic tubes without an anticoagulant but immediately centrifuged to remove cells and platelets; we refer to such sample as plasma in this paper. Although some clotting activity may take place before and during centrifugation of the blood sample, our experiments indicate that activation of coagulation factors, e.g., FVII, is very limited (see Figure 5A). To measure remaining hPEPD, the incubated samples were cleared of potential fibrin clots by centrifugation and then analyzed by SDS-PAGE and silver staining (using the LC 6070 kit from Invitrogen), IB or measurement of hPEPD enzymatic activity. hPEPD enzymatic activity was measured using glycyl-proline as a substrate as previously described [60]. The precipitates from each incubated sample were washed with PBS twice and then resuspended in 2.5% SDS (20 μl per sample); each solution was mixed with 10 μl loading dye and analyzed for hPEPD by IB, using pure hPEPD as a standard and factor XIIIa (FXIIIa) as a binding control, respectively.

To measure degradation of hPEPD in plasma samples with or without removal of FVII and FVIIa, plasma samples from WT mice as described above were either used directly or immunodepleted of FVII and FVIIa before use. To remove FVII and FVIIa from plasma, 500 μl plasma was incubated with an antibody specific for FVII/FVIIa (GTX79785; 10 μg) and protein A sepharose beads for 1 h at RT. Following centrifugation at 13,000 x g for 1 min at 4°C, the supernatant fraction was collected and analyzed by IB to confirm depletion of FVII and FVIIa. Next, 9.2 pmol of hPEPD was incubated with 100 μl of regular plasma or the plasma depleted of FVII/FVIIa, with or without FIIa (10 pmol) for 24 h at RT. Potential fibrin clots formed during the incubation was removed by centrifugation at the end of the incubation, and remaining hPEPD was measured by hPEPD enzymatic activity analysis.

To measure degradation of mSRC or hAβ1-42 by FVIIa, mSRC (0.17 μM) or hAβ1-42 (2.2 μM) was incubated with or without FVIIa (10 nM) and TF (10 nM) in PBS in a total volume of 100 μl containing 5 mM CaCl_2_ for desired times at RT. All samples after incubation were resolved by SDS-PAGE and stained by silver. In the case of Aβ1-42 which aggregated during the incubation, the aggregates were re-dissolved in 30 μl of 2% SDS and combined with the supernatant fraction before SDS-PAGE.

### Measurement of binding of hPEPD, mSRC or hAβ1-42 to a coagulation factor or its mutant

Binding reactions were carried out in PBS in a total volume of 100 μl for 2 h at 37°C. To assess binding of hPEPD to HMWK, PK, FXI, FXII or a FXII mutant, hPEPD at 40 nM was incubated with a potential binding partner at 0.5 μM. All FXII mutants are His tagged, while FXII is either 6XHis-tagged (generated in our own laboratory) or not His-tagged (purchased commercially), depending on the experiment. To compare hPEPD and its mutants for binding to FXII, hPEPD or a mutant (40 nM) was incubated with FXII at 0.5 μM. Likewise, to assess binding of mSRC to FXII and its mutants, mSRC (40 nM) was incubated with FXII or a mutant at 0.5 μM. At the end of the incubation, 300 μl PBS containing a specific primary antibody was added to the incubation solution, which was further incubated at 4°C overnight, followed by IP by protein G-agarose, and the precipitates were analyzed by IB. The above approach was not used to assess binding of hAβ1-42 to FXII and its mutants, due to the tendency of hAβ1-42 to aggregate. Instead, hAβ1-42 (200 nM) was incubated with FXII or a mutant (20 nM) in PBS in a total volume of 100 μl for 2 h at RT; the solution was then centrifuged at low speed to separate the supernatants from the precipitates. The precipitates were re-dissolved in 30 μl of 2% SDS, which were analyzed along with the supernatants by IB.

### Measurement of FXII activation

FXII activation by hPEPD, its mutants, mSRC or hAβ1-42 *in vitro* was measured by a chromogenic assay as described previously [36]. hPEPD and its mutants were each evaluated at 40 and 400 nM. hAβ1-42 was evaluated at 0.05, 0.5 and 11.1 μM. mSRC was evaluated at 20 and 200 nM. FXII activation was followed for 3 h at RT by monitoring the conversion of the chromogenic substrate at 405 nm by a microtiter plate reader. Notably, the assay specifically measures FXII activation, as omission of FXII from the reaction solution abrogated conversion of the chromogenic substrate in all reactions in our experiments.

### Statistical analysis

Data were analyzed by analysis of variance (ANOVA), followed by Tukey multiple comparisons test. For data that are highly skewed, log transformation was performed before ANOVA, as indicated in figure legend. P value of 0.05 or lower was considered statistically significant

## SUPPLEMENTARY FIGURES AND TABLES


