# Supplementary material for: A plasma proteolysis pathway comprising blood coagulation proteases
Source: Oncotarget. 2016 Feb 7;7(27):40919–38. doi: 10.18632/oncotarget.7261 (PMC5173032; doi:10.18632/oncotarget.7261)
Supplement: Supplementary file 1 [file oncotarget-07-40919-s001.pdf]

A plasma proteolysis pathway comprising blood coagulation proteases

Supplementary Materials and Methods

Materials

Human coagulation factors FX (HCX-0050) and FII (HCP-0010) were purchased from Haematologic Technologies.

Preparation of Mouse PEPD (mPEPD)

mPEPD was obtained from the kidneys of C57BL/6 mice by immunoaffinity purification. First, a PEPD antibody was covalently linked to protein A-sepharose beads. Five μg of anti-PEPD (Ab111851, Abcam) was incubated with 50 μl of 6 mg/ml protein A-sepharose beads (17-6002-35, GE Healthcare Life Sciences) at RT for 1 h. The antibody-bound beads were washed three times with PBS and incubated with cross linker BS3 (Pierce) at 2 mM for 30 min (RT). The cross-linking reaction was terminated by adding 50 mM Tris to the mixture (final, pH 7.5), followed by incubation at RT for 15 min. The antibody-bound beads were washed three times with PBS, and the beads from each cross-linking reaction was incubated in 1 ml of blocking buffer containing 100 mM ethanolamine (pH 8.2) at RT for 15 min, in order to block any remaining NHS-ester groups on BS3, which was followed by washing the bead complexes with PBS three times. Next, kidneys from 8-week-old C57BL/6 mice were minced with scissors and homogenized to 10 times the volume (v/w) in ice-cold 50 mM Tris-HCl (pH 7.4) with a Dounce homogenizer. The homogenates were centrifuged at 9000 x g for 20 min (4 ^{o}C) to remove tissue debris. The supernatant sample was incubated with the PEPD antibody-protein A-sepharose bead conjugates (0.5 ml sample with 50 μl beads) overnight at 4 ^{o}C. The beads were then washed with 50 mM Tris (pH 8.0) three times, and the bound mPEPD molecules were eluted with 1 ml of 100 mM glycine-HCl (pH 2.8) four times. The eluates were pooled, and the pH of

the solution was adjusted to approximately 7.4, using 1 M Tris-HCl (pH 9.5). The mPEPD solution was then concentrated using Ultracel YM-30 Centricon (Millipore). Protein concentration in the sample was measured by the BCA protein assay kit. The mPEPD preparation was checked by western blotting, and its purity was confirmed by SDS-PAGE, followed by silver staining (Supplementary Fig. 1A and B), using hPEPD for comparison.

Measurement of Potential Activation of FX, FII or FVII by hPEPD

hPEPD (40 nM) was incubated with FX, FII or FVII (0.5 μM) in PBS at RT in the presence of 5 mM $CaCl_2$ for 24 h, followed by IB analysis for potential activation/cleavage of each coagulation factor. FXa, FIIa and FVIIa were used as positive controls in the experiments.

Measurement of Plasma Levels of Aspartate Transaminase (AST) and Alanine Transaminase (ALT)

Plasma AST activity was measured using the InfinityTM AST (GOT) Reagent kit (Thermo, TR70121). Plasma ALT activity was measured using the InfinityTM ALT (GPT) Reagent kit (Thermo, TR71121). Each assay was performed according to the manufacturer’s instruction.

Supplementary Data

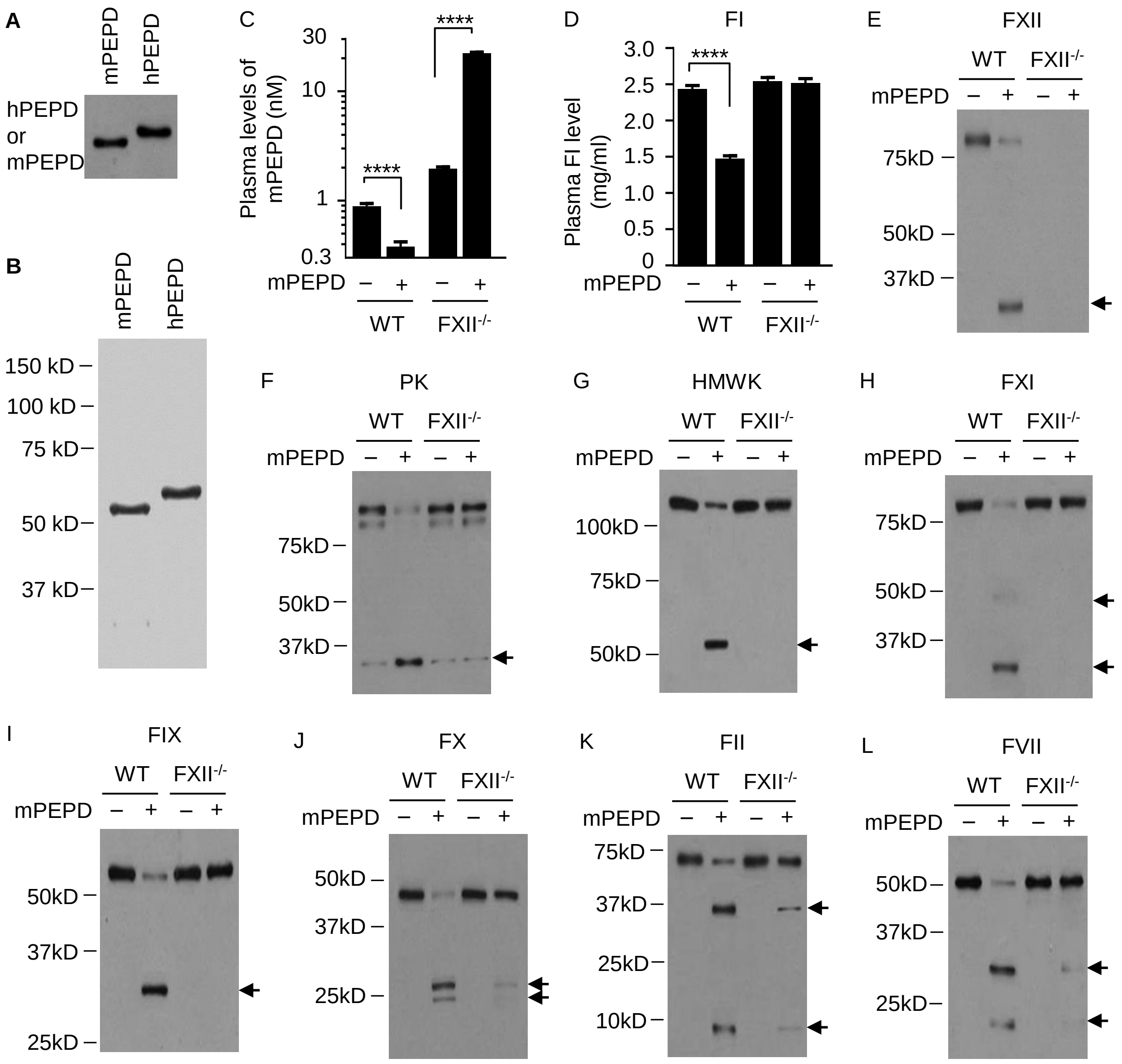

Supplementary Fig. 1. Characterization of mPEPD, and its activation of the proteolysis pathway. (A) mPEPD, measured by IB and compared to hPEPD. (B) mPEPD, measured by SDS-PAGE followed by silver staining and compared to hPEPD. (C, D) Plasma levels of mPEPD and fibrinogen (FI) in mice treated with mPEPD, measured by ELISA. Error bars indicate SD (n=3). Data were analyzed by two-way ANOVA, followed by Tukey multiple comparisons test. Data in C were log transformed before ANOVA. **** P<0.0001. (E-L) Effect of mPEPD on plasma coagulation factors in mice, measured by IB. Plasma samples were obtained from WT mice and FXII$^{-/-}$ mice at 6 h after i.p. injection of solvent or mPEPD (0.2 mg/kg). Each lane represents 7.5 μl of plasma sample. Arrows indicate cleaved fragments.

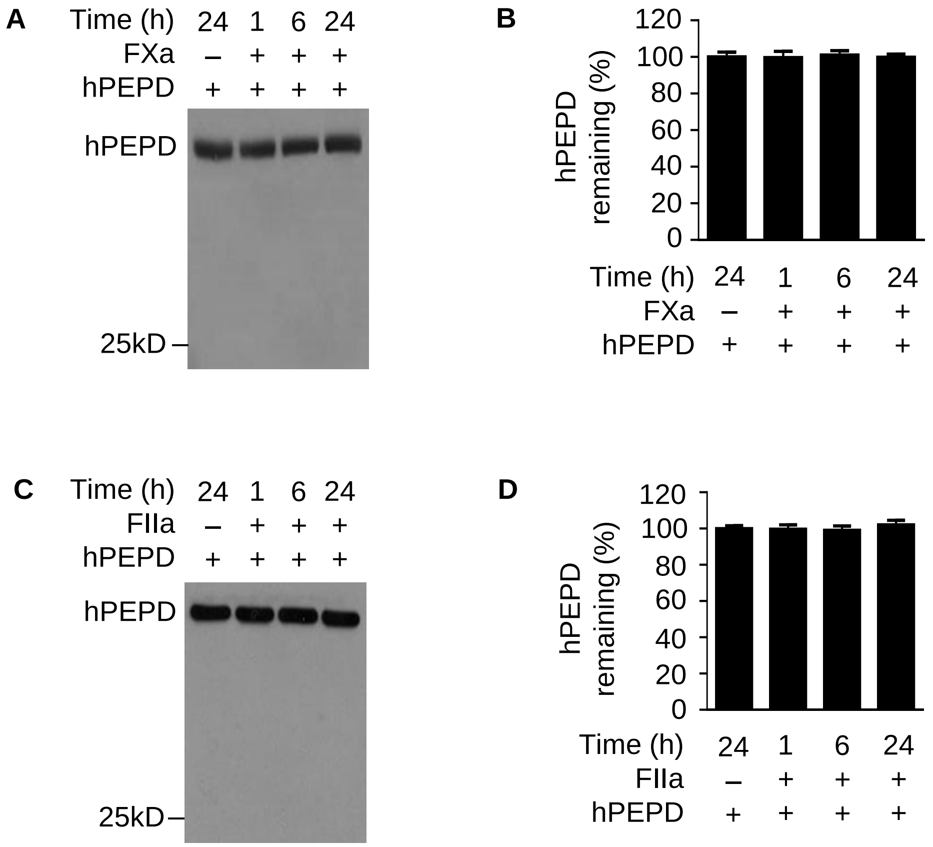

Supplementary Fig. 2. No effect of FXa and FIIa on hPEPD stability. hPEPD (90 nM) was incubated alone, with FXa (100 nM) or FIIa (100 nM) in PBS containing 5 mM $CaCl_2$ (total volume of 0.1 ml) for a specific time at RT. The incubated samples were analyzed for remaining hPEPD by IB (A, C) or by measurement of hPEPD enzymatic activity (B, D). Error bars indicate SD (n=3).

hPEPD (493 amino acids) and its mutants

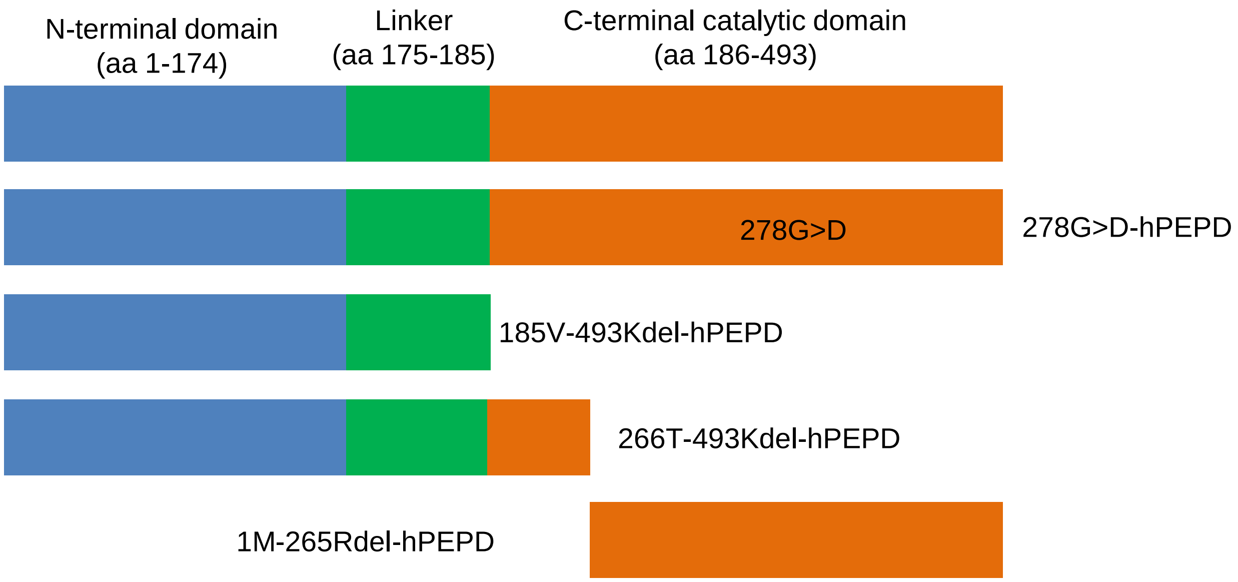

Supplementary Fig. 3. Sequence information of hPEPD and its mutants.

Each protein has 6xHis tagged to its carboxy terminus.

A Location of various domains in human FXII (615 amino acids):

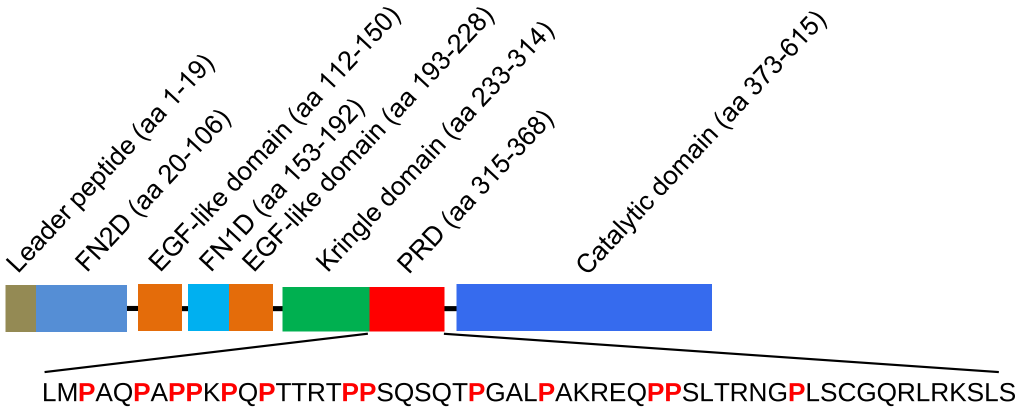

B Human FXII mutants:

20I-50Pdel-FXII: deletion of residues #20-50 known to bind to negatively charged surface

153T-172Rdel-FXII: deletion of residues #153-172 known to bind to negatively charged surface

315L-368Sdel-FXII: deletion of residues #315-368

6Psdel-FXII: deletion of first 6 prolines in the PRD

13Ps>13As-FXII: converting all 13 prolines in the PRD to alanines

C

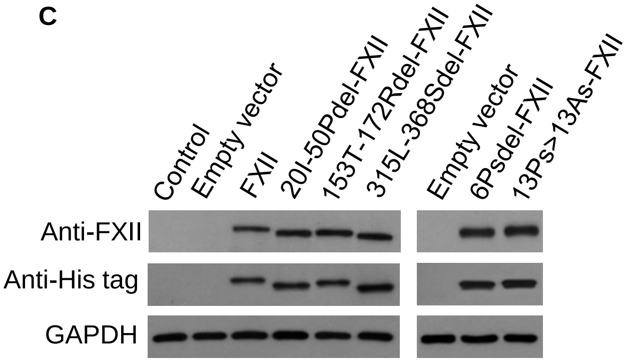

D

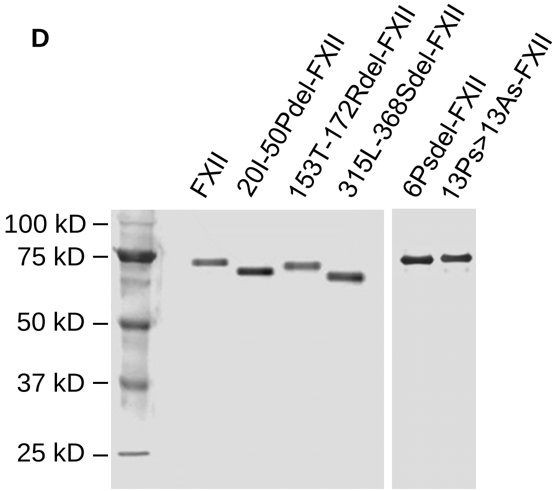

Supplementary Fig. 4. Characterization of FXII and its mutants. (A) Location of various domains in human FXII. (B) Sequence information on FXII mutants. (C) Comparison of relative molecular size of recombinant FXII and its mutants. FXII and its mutants were generated in CHO-K1 cells, purified by NI-NTA agarose chromatography, and compared for molecular size by IB, using an antibody binding to either their C-termini or C-terminal His tag. (D) Purified FXII and its mutants were resolved by SDS-PAGE and stained by silver to assess purity.

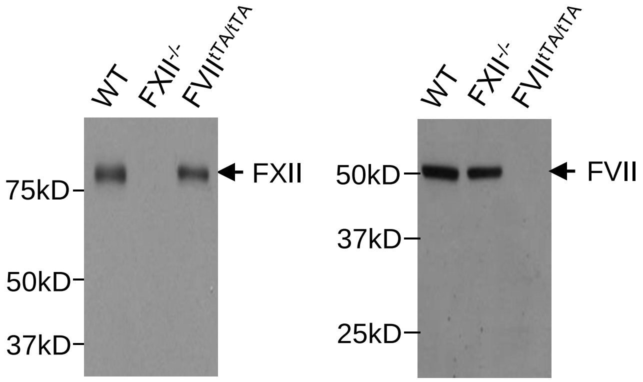

Supplementary Fig. 5. Plasma levels of FXII and FVII in WT mice, FXII$^{-/-}$ mice and FVII$^{tTA/tTA}$ mice. Plasma samples (7.5 μl each) were analyzed by IB.

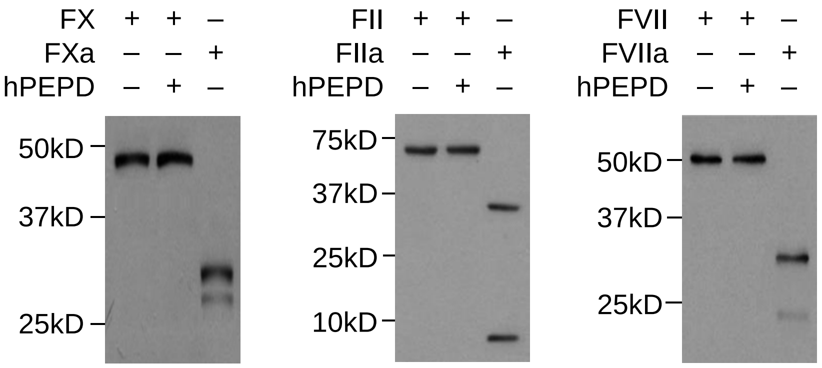

Supplementary Fig. 6. hPEPD does not directly activate FX, FII or FVII. hPEPD (40 nM) was incubated with FX, FII or FVII (0.5 μM for each factor) in the presence of 5 mM $CaCl_2$ in PBS at RT for 24 h and then analyzed by IB. FXa, FIIa and FVIIa were used as positive controls in the experiments.

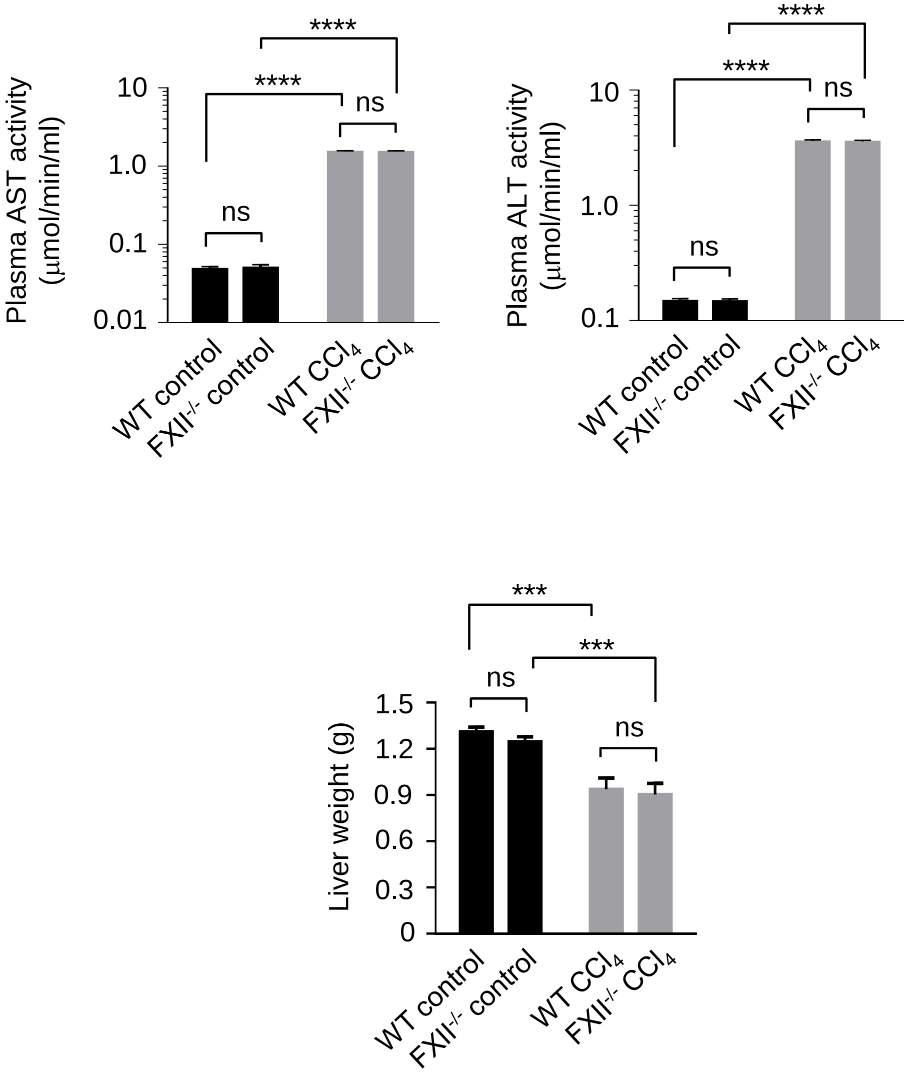

Supplementary Fig. 7. Changes in plasma levels of AST and ALT and in liver weight after treatment with CCl_4. WT mice and $FXII^{-/-}$ mice were treated i.p. with vehicle or CCl_4 at 0.5 g/kg; 24 h later, the mice were killed, plasma samples were prepared and measured for AST and ALT activities, and the livers were weighed. Error bars indicate SD (n=3). The data were analyzed by two-way ANOVA, followed by Tukey multiple comparisons test. *** P<0.001; **** P<0.0001; ns, not significant.

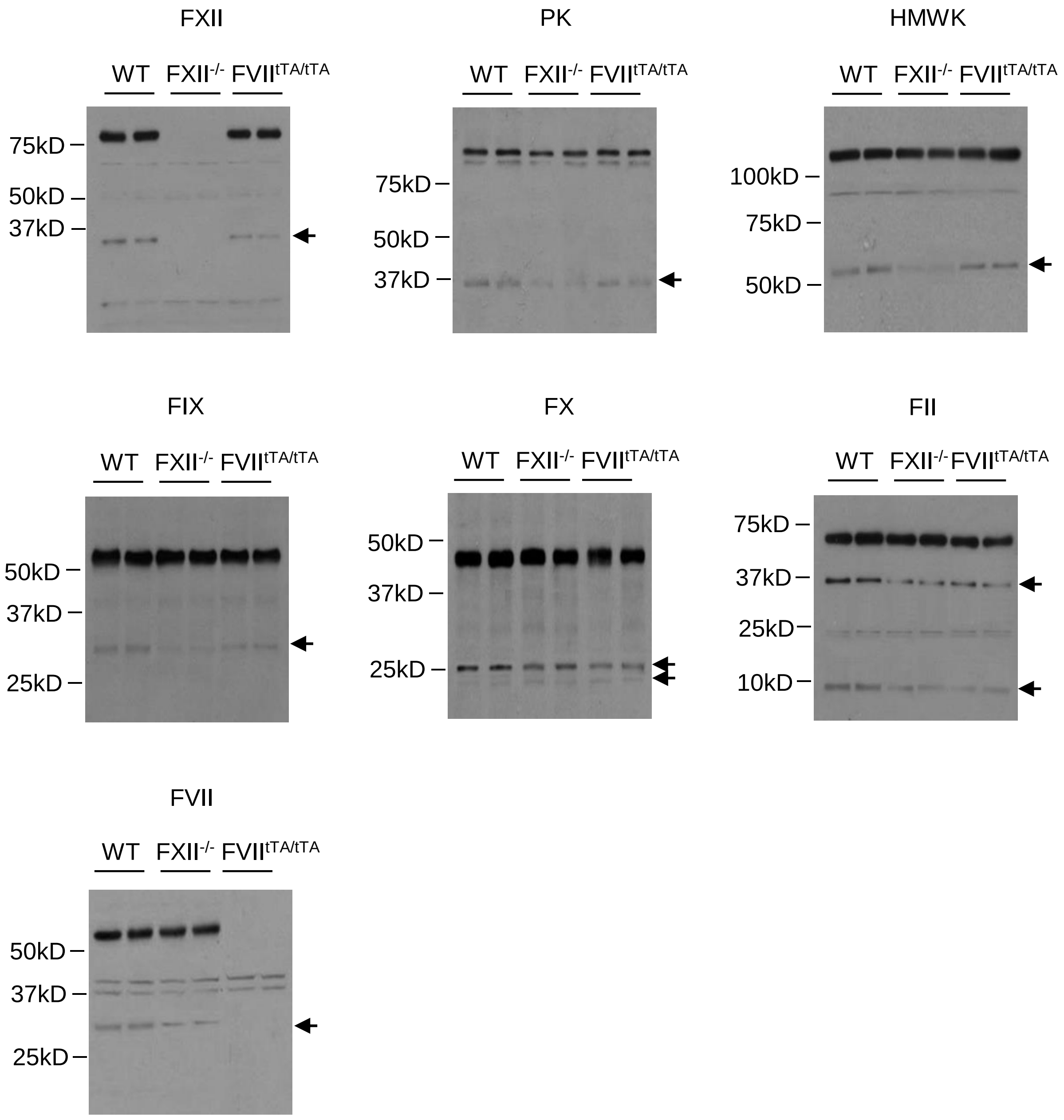

Supplementary Fig. 8. Background activation of the FXII-FVII pathway factors. Plasma samples (7.5 μl each) from untreated WT mice, $FXII^{-/-}$ mice and $FVII^{tTA/tTA}$ mice were analyzed by IB. Each lane represents one mouse. Note: All the films were highly overexposed in order to detect the minute levels of cleaved fragment(s) of each factor. Arrows indicate cleaved fragments

Supplementary Table 1. The sequences of primers used for cloning, site-directed mutations and partial deletions of His-tagged human FXll

Target vector	Primer	Sequence
pCMV6-XL5-FXll	For	5’-GAATTCgaccaacggacggacgc-3’
	Rev	5’-GTCGAC ggaaacggtgtgctccc-3’
pCMV6-XL5-FXll-6XHis (C-terminal 6XHis insertion)	For	5’-tagattgcggccgcggcatcatcaccatcaccattaatcatagctgtttcctg-3’
	Rev	5’-caggaaacagctatgattaatggtgatggtgatgatgccgcggccgcaatcta-3’
pCMV6-XL5-20I-50Pdel-FXll-6XHis	For	5’-ggagtcaacactttcgttccagtaccaccggc-3’
	Rev	5’-gccggtggtactggaacgaaagtgttgactcc-3’
pCMV6-XL5-153T-172Rdel-FXll-6XHis	For	5’-aagaatgagatatggtatagactggccagccaggc-3’
	Rev	5’-gcctggctggccagtctataccatatctcattctt-3’
pCMV6-XL5-315L-368Sdel-FXll-6XHis	For	5’-tcccctaggcttcatgtcccatcgatgacccg-3’
	Rev	5’-cgggtcatcgatgggacatgaagcctagggga-3’
pCMV6-XL5-6Psdel-FXll-6XHis	For	5’-atgtcccactcatgacgacccggacccc-3’
	Rev	5’-ggggtccgggtcgtcatgagtgggacat-3’
pCMV6-XL5-13Ps>13As-FXll-6XHis[a]	For 1	5’-catggccgcgcaggcggcagcggcgaaggctcaggccac-3’
	For 2	5’-cgacccggaccgcggctcagtcccag-3’
	For 3	5'-cagtcccagaccgcgggagccttgc-3'
	For 4	5'-cgggagccttggcggcgaagcgg-3'
	For 5	5'-aagcgggagcaggcggcttccctgacca-3'
	For 6	5'-accaggaacggcgcactgagctgcg-3'

[a] Multiple sets of primers were used to generate this FXII mutant.
